# Long-term climate data description in Ethiopia

**DOI:** 10.1016/j.dib.2017.07.052

**Published:** 2017-07-29

**Authors:** Gezahegn Abebe

**Affiliations:** Department of Sociology and Human Geography, University of Oslo, Norway

**Keywords:** Erratic rainfall, Temperature, Farm inputs, Cereals, Consumer price, Ethiopia

## Abstract

This article presents long-term analyzed rainfall and temperature data obtained from the National Metrological Agency (NMA) of Ethiopia. Using tables and graphic trends of analysis, the article shows the low and declining level of average annual rainfall as well as the high inter-annual fluctuations for 18 weather stations located in different agro-climatic zones of the country. The high variation of annual maximum and minimum temperature has been similarly observed for decades in the stations. Ethiopia's average annual temperature has risen between 1955 and 2015 by 1.65 °C. The country's agricultural production depends heavily on local temperature and rainfall. The evidence is clear that a slight change in such climatic elements negatively affects the food security condition of both producers and consumers. Although data from the Central Statistical Agency (CSA) show that major cereal crop production has increased at the national level, partly due to the increasing application of fertilizers and modern seeds, Ethiopia's food security condition is deteriorating due to global climatic events caused droughts and rain failure. The rate of food price inflation is thus often higher than the general consumer price inflation rate.

**Specification Table**

**Table:** 

Subject area	Environmental studies
More specific subject area	Climate change
Type of data	Figures and tables
How data was acquired	Climate data were obtained following formal application procedure to the authority. Different year agricultural sample survey and the consumer price index data available at the CSA of Ethiopia were used.
Data format	Analyzed
Data source location	18 weather stations: *Addis Ababa; Arba Minch; Axum; Bahir Dar; Beshoftu; Combolcha; Debre Markos; Dire Dawa; Gode; Gondar; Gore; Hawassa; Jimma; Mekele; Methara; Neghele; Nekemte; and Robe.*
Experimental factors	Data used in this article were obtained from the NMA and CSA of Ethiopia.
Experimental features	Tables and graphic trends of analysis were employed.
Data accessibility	The data are with this article.

**Value of the data**

• Gives information on the changing condition of climatic elements' impact on production and food prices.
• Can be reproduced by researchers and experts working in the field.
• Useful to identify vulnerable communities and social groups to the effects of climate change risk for interventions.

## 1 Data

**Fig. 1:**
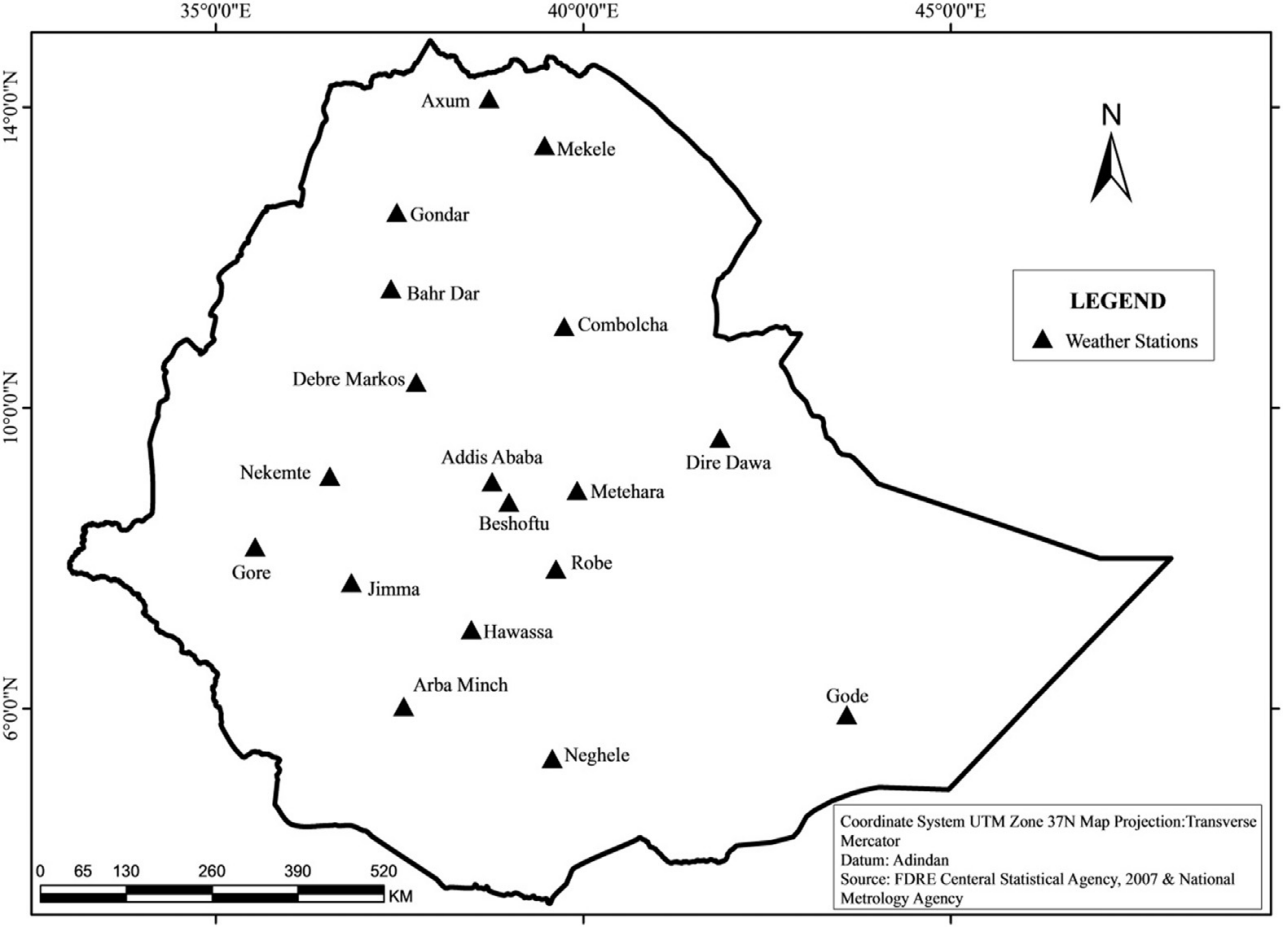
Location map of 18 weather stations.

**Fig. 2:**
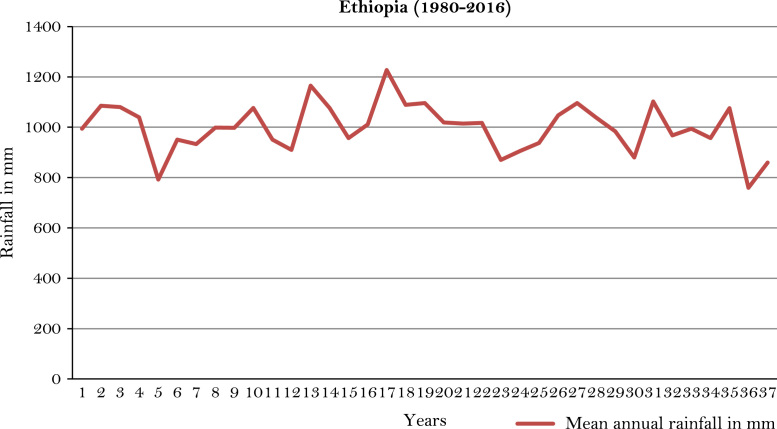
Mean annual rainfall of Ethiopia.*Source*: Computed based on raw data from National Metrological Agency (NMA) of Ethiopia.

**Fig. 3:**
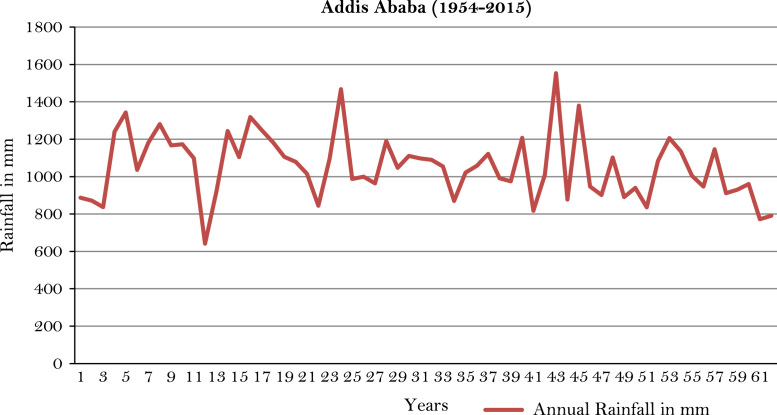
Annual rainfall in *Addis Ababa* weather station.*Source*: Computed based on raw data from National Metrology Agency (NMA) of Ethiopia.

**Fig. 4:**
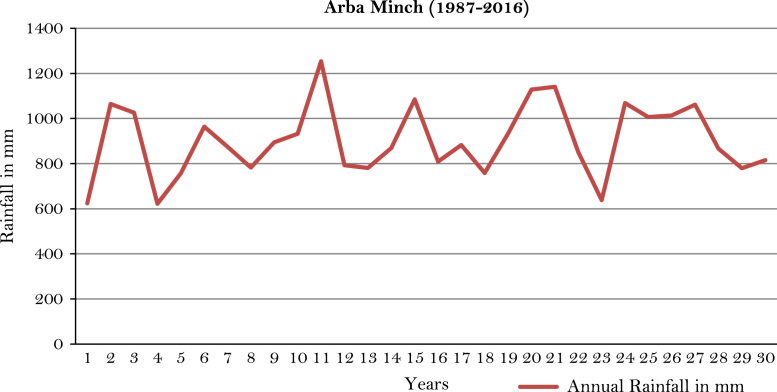
Annual rainfall in *Arba Minch* weather station.*Source*: Computed based on raw data from National Metrology Agency (NMA) of Ethiopia.

**Fig. 5:**
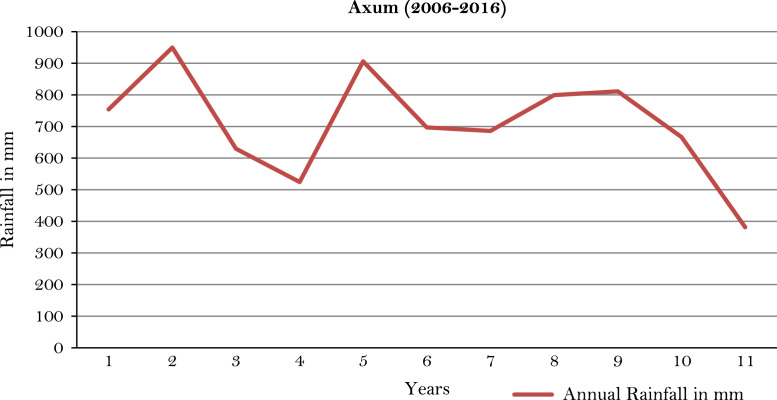
Annual rainfall in *Axum* weather station.*Source*: Computed based on raw data from National Metrology Agency (NMA) of Ethiopia.

**Fig. 6:**
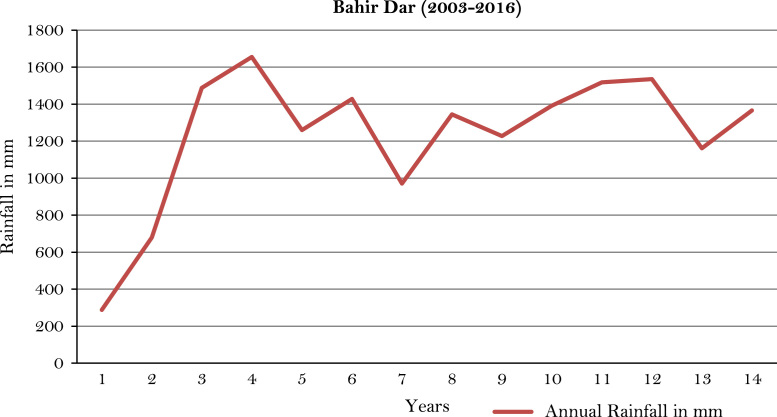
Annual rainfall in *Bahir Dar* weather station.*Source*: Computed based on raw data from National Metrology Agency (NMA) of Ethiopia.

**Fig. 7:**
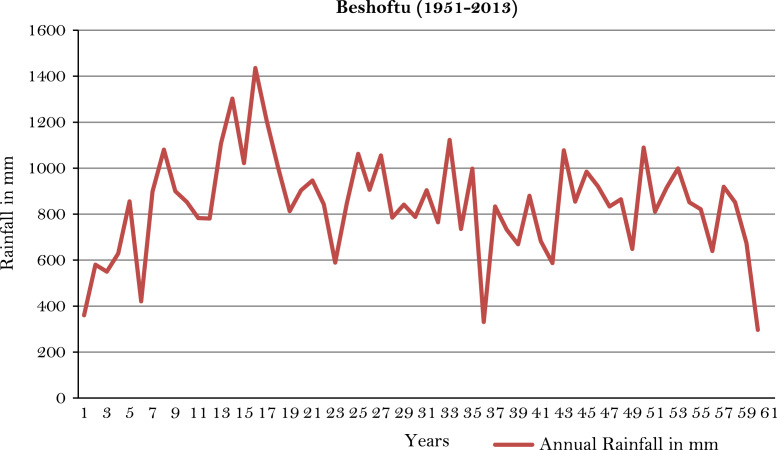
Annual rainfall in *Beshoftu* weather station.*Source*: Computed based on raw data from National Metrology Agency (NMA) of Ethiopia.

**Fig. 8:**
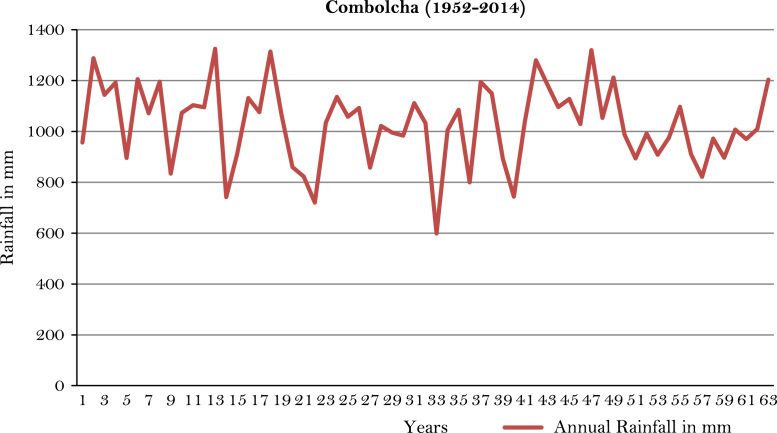
Annual rainfall in *Combolcha* weather station.*Source*: Computed based on raw data from National Metrology Agency (NMA) of Ethiopia.

**Fig. 9:**
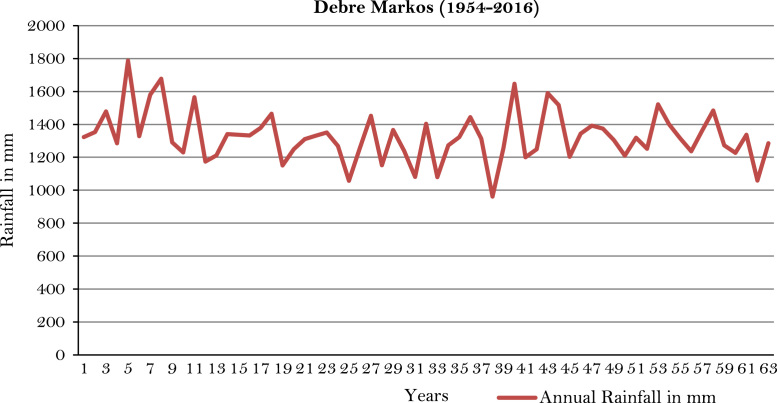
Annual rainfall in *Debre Markos* weather station.*Source*: Computed based on raw data from National Metrology Agency (NMA) of Ethiopia.

**Fig. 10:**
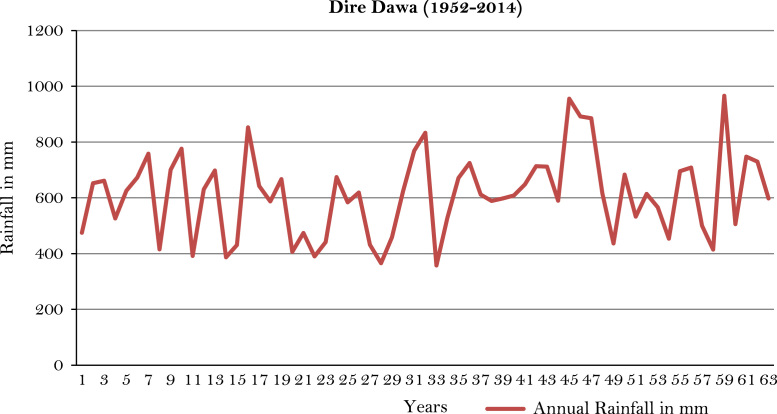
Annual rainfall in *Dire Dawa* weather station.*Source*: Computed based on raw data from National Metrology Agency (NMA) of Ethiopia.

**Fig. 11:**
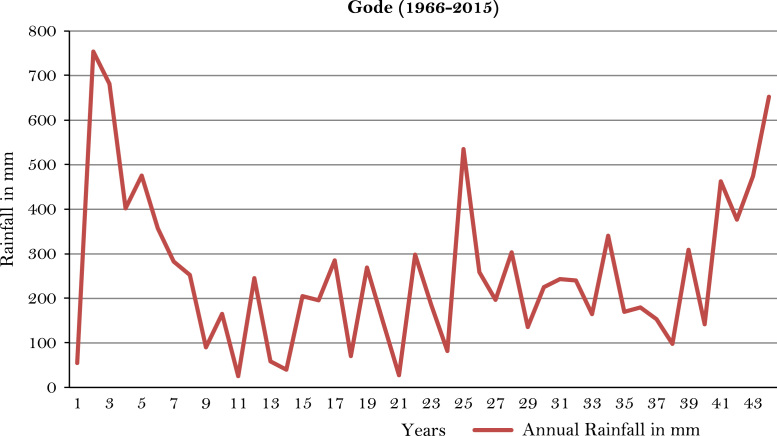
Annual rainfall in *Gode* weather station.*Source*: Computed based on raw data from National Metrology Agency (NMA) of Ethiopia.

**Fig. 12:**
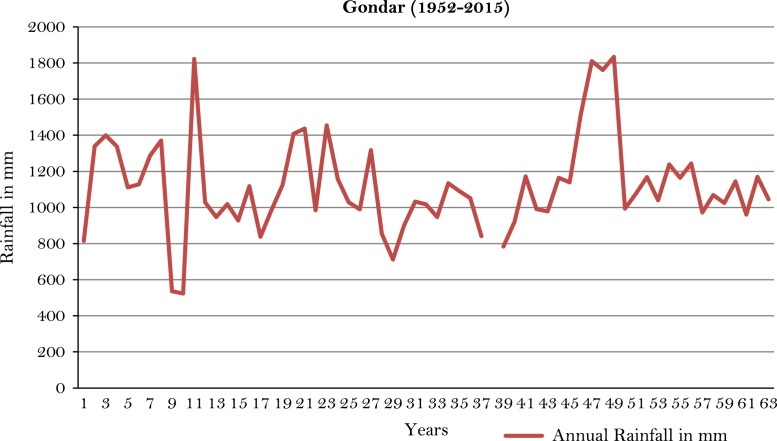
Annual rainfall in *Gondar* weather station.*Source*: Computed based on raw data from National Metrology Agency (NMA) of Ethiopia.

**Fig. 13:**
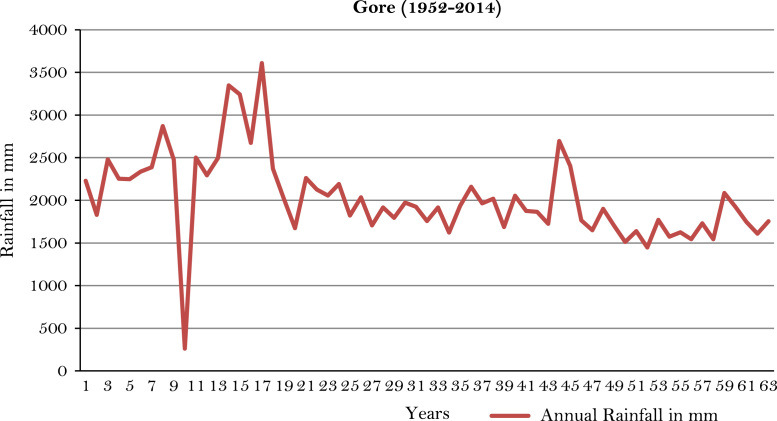
Annual rainfall in *Gore* weather station.*Source*: Computed based on raw data from National Metrology Agency (NMA) of Ethiopia.

**Fig. 14:**
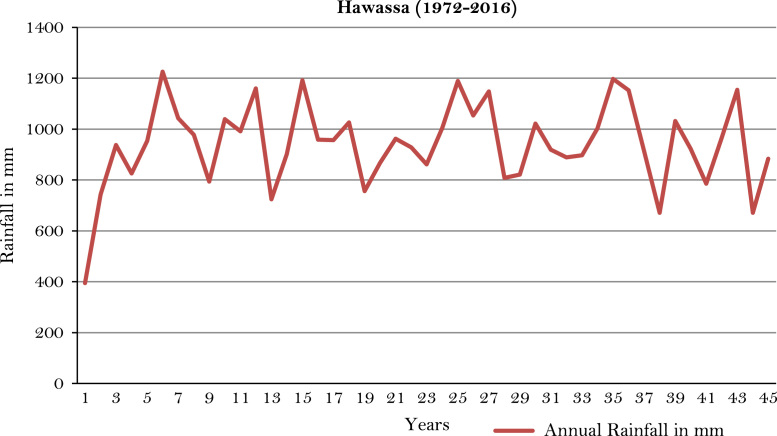
Annual rainfall in *Hawassa* weather station.*Source*: Computed based on raw data from National Metrology Agency (NMA) of Ethiopia.

**Fig. 15:**
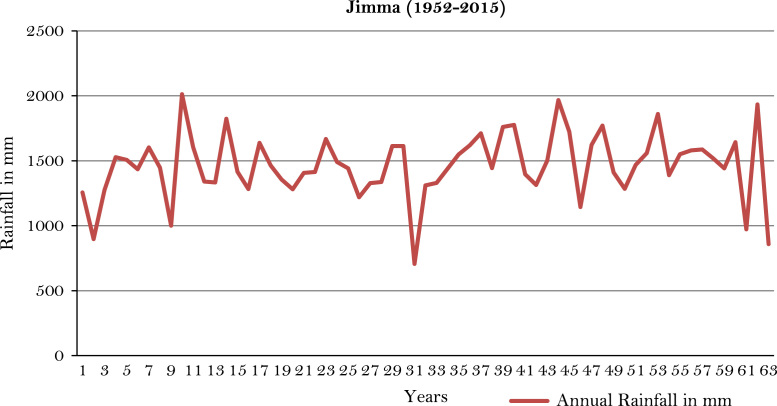
Annual rainfall in *Jimma* weather station.*Source*: Computed based on raw data from National Metrology Agency (NMA) of Ethiopia.

**Fig. 16:**
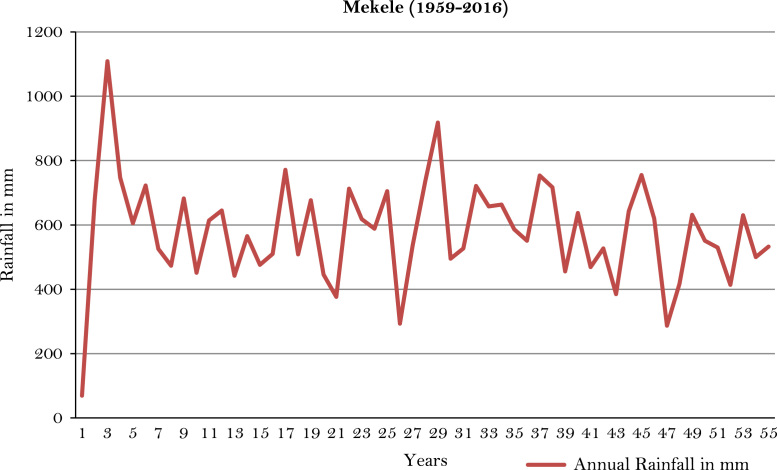
Annual rainfall in *Mekele* weather station.*Source*: Computed based on raw data from National Metrology Agency (NMA) of Ethiopia.

**Fig. 17:**
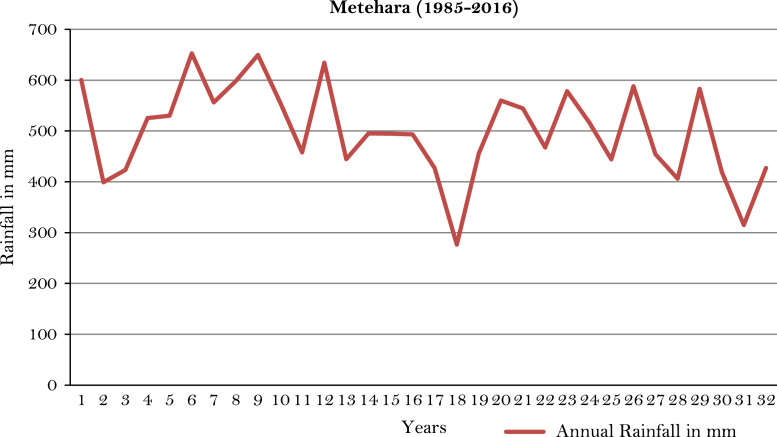
Annual rainfall in *Metehara* weather station.*Source*: Computed based on raw data from National Metrology Agency (NMA) of Ethiopia.

**Fig. 18:**
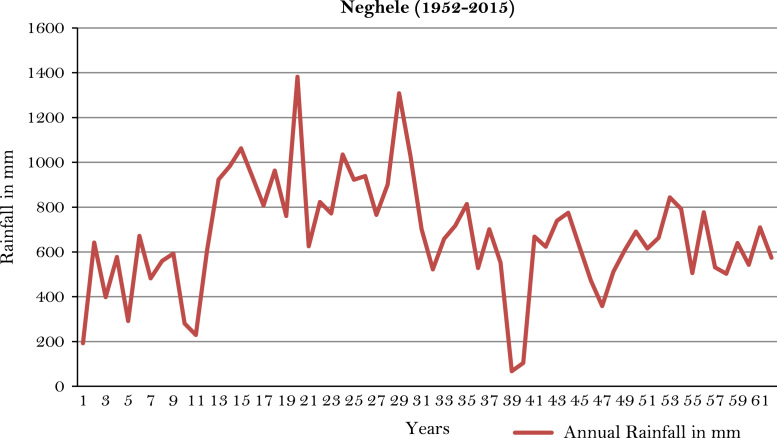
Annual rainfall in *Neghele* weather station.*Source*: Computed based on raw data from National Metrology Agency (NMA) of Ethiopia.

**Fig. 19:**
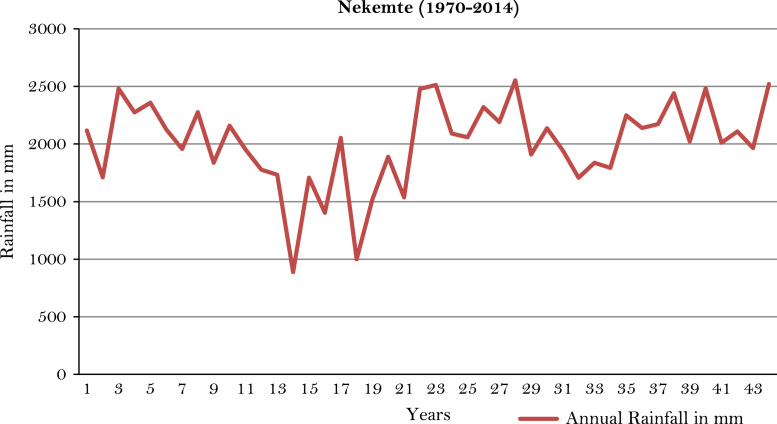
Annual rainfall in *Nekemte* weather station.*Source*: Computed based on raw data from National Metrology Agency (NMA) of Ethiopia.

**Fig. 20:**
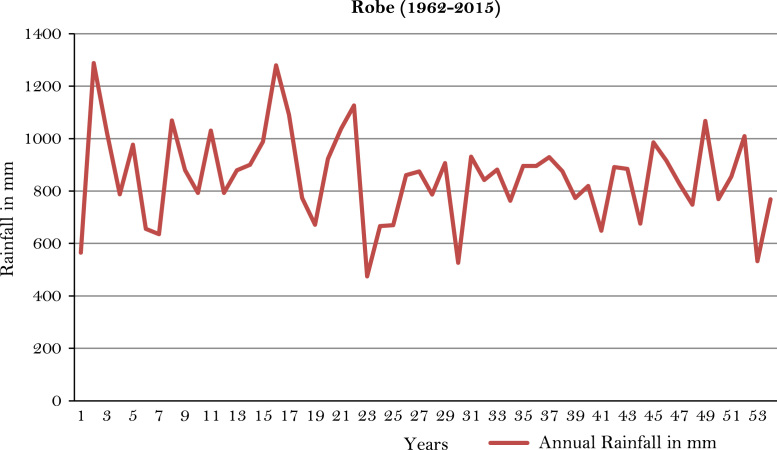
Annual rainfall in *Robe* weather station.*Source*: Computed based on raw data from National Metrology Agency (NMA) of Ethiopia.

**Fig. 21:**
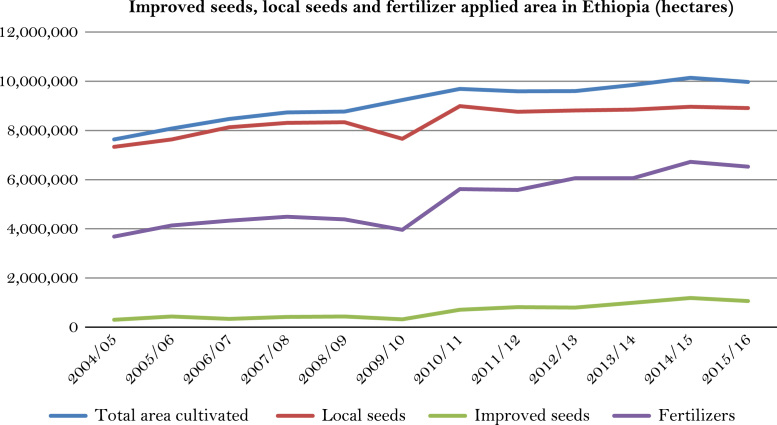
Area cultivated under improved seeds, local seeds and fertilizer for cereals only in Ethiopia.*Source*: Computed based on raw data from Central Statistical Agency (CSA) of Ethiopia.

**Fig. 22:**
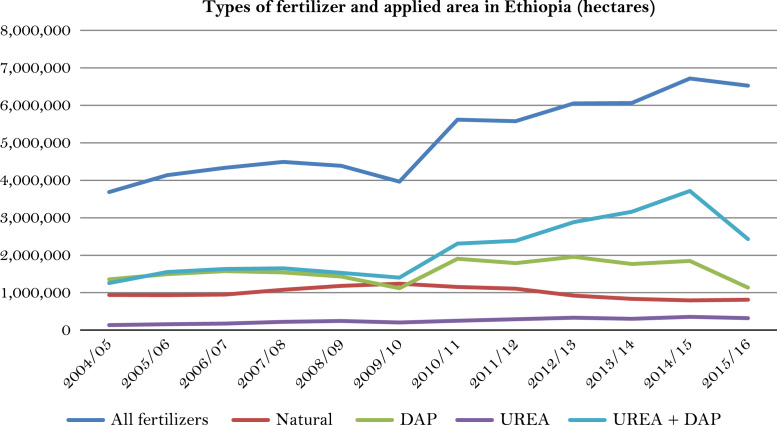
Fertilizer applied area and types of fertilizer for cereals only in Ethiopia.*Source*: Computed based on raw data from Central Statistical Agency (CSA) of Ethiopia.

**Fig. 23:**
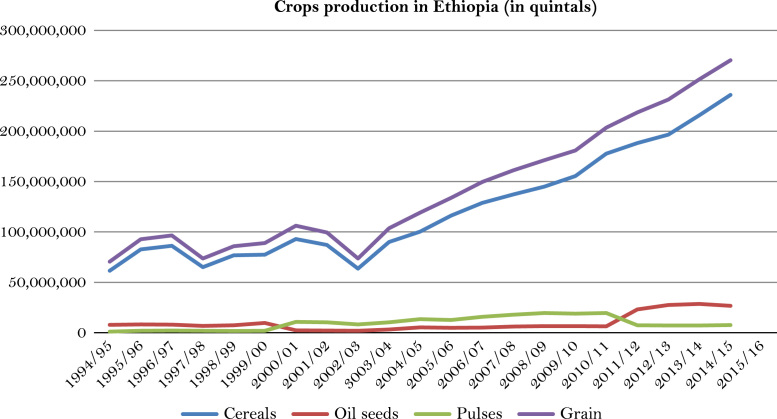
Major crops production in Ethiopia (1994/95–2014/15).*Source*: Computed based on raw data from Central Statistical Agency (CSA) of Ethiopia. *Note:* Grain refers to all cereals, pulses and oil seeds. 1 quintal=100 kg.

**Fig. 24:**
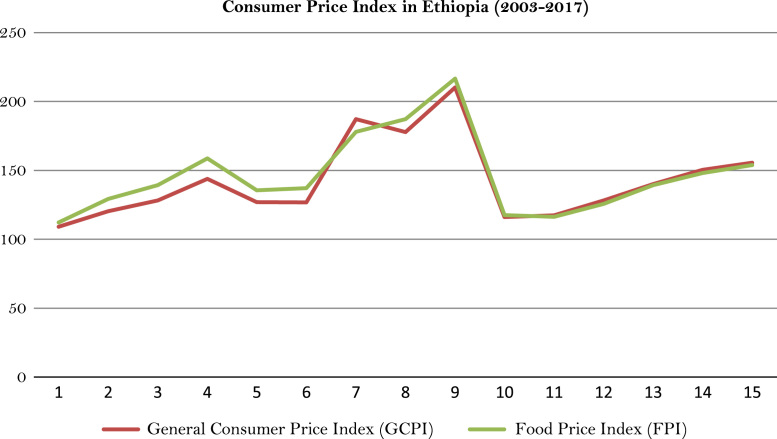
Consumer Price Index in Ethiopia.*Source*: Computed based on raw data from Central Statistical Agency (CSA) of Ethiopia.

The figures and tables of rainfall and temperature were analyzed based on the data obtained from 18 weather stations located in different agro-climatic zones of Ethiopia. Fig. 1 is the location map of metrological stations. The declining and low level of average annual rainfall overtime as well as high inter-annual fluctuation for 18 weather stations are presented in Fig. 2, Fig. 3, Fig. 4, Fig. 5, Fig. 6, Fig. 7, Fig. 8, Fig. 9, Fig. 10, Fig. 11, Fig. 12, Fig. 13, Fig. 14, Fig. 15, Fig. 16, Fig. 17, Fig. 18, Fig. 19, Fig. 20. Information on temperature are presented in Tables. Table 1 shows the average annual temperature of Ethiopia (1980–2016). The mean annual temperature of Ethiopia is presented in Table 2. The following tables (Table 3, Table 4, Table 5, Table 6, Table 7, Table 8, Table 9, Table 10, Table 11, Table 12, Table 13, Table 14, Table 15, Table 16, Table 17, Table 18, Table 19) present the variation of mean annual maximum and minimum temperatures of the weather stations. In Fig. 21, Fig. 22 area cultivated under improved seeds, local seeds and use of fertilizers and types of fertilizers for cereals crop only are presented. The last two Fig. 23, Fig. 24 demonstrate the progressive increase in agricultural production such as cereals, oil seeds and pulses and the consumer price index respectively.

**Table 1:** Average annual temperature in Ethiopia: representative weather stations.

**Year**	Addis Ababa	Arba Minch	Hawassa	Combolcha	Debre Markos	Debre Zeit	Dire Dawa	Robe	Gode Metekel	Gondar	Gore	Jimma	Mekele	Metehara	Neghele	Nekemte	**Average annual Temp.**
**1955**	18.9	–	–	17.69	15.24	18.55	24.86	–	–	18.65	17.85	18.54	–	–	18.37	–	**18.70**
**1960**	18.85	–	–	19.7	15.2	18.87	24.97	12.72	–	18.37	18.17	19.3	17.62	–	19.66	–	**18.49**
**1965**	18.9	–	–	18.89	15.28	19.09	24.7	12.19	27.98	19.59	17.73	18.82	18.57	–	19.11	–	**19.23**
**1970**	19.65	–	–	19.2	16.45	19.07	24.93	12.93	28.82	19.9	18.24	18.73	18.63	–	19.38	17.25	**19.47**
**1975**	19.82	–	18.68	17.82	15.76	18.26	25.02	12.82	28.86	20.03	17.9	18.6	16.99	–	19.17	17.06	**19.05**
**1980**	19.92	–	19.9	19.07	16.22	18.93	25.4	14	28.92	19.97	18.6	19.19	17.66	–	20.05	18.06	**19.70**
**1985**	15.72	24.62	18.74	15.82	16.06	18.38	25.17	15.12	28.05	20.16	18.1	18.83	17.21	24.89	19.12	17.65	**19.60**
**1990**	16.1	24.08	19.76	19.73	16.04	19.13	25.37	15.02	29	19.54	19.95	19.01	11.2	25.37	21.23	18.51	**19.94**
**1995**	16.6	23.8	20.2	19.49	16.95	19.65	25.85	14.93	–	20.85	19.17	19.94	17.88	26.17	21.12	18.75	**20.30**
**2000**	16.62	23.66	19.88	19.3	16.35	19.41	25.68	14.08	28.91	19.44	18.82	19.79	18.15	25.42	20.75	18.6	**20.69**
**2005**	16.89	23.88	20.21	19.73	16.83	18.48	26.06	15.2	29.72	20.67	19.56	19.6	18.01	25.93	21.28	19.0	**20.56**
**2010**	16.95	23.94	20.62	20.4	17.09	18.78	25.9	15.43	30	20.73	19.42	20.14	18.23	26.07	21.76	18.99	**20.75**
**2015**	18.55	24.65	21.44	20.48	17.37	20.18	25.71	15.72	29.67	20.67	19.36	20.43	18.23	26.88	22.44	18.57	**20.35**

**Table 2:** Variation of annual maximum and minimum temperature in *Addis Ababa* (1955–2015).

	**Year**	**Average annual temp. (Max)**	**Average annual temp. (Min)**	**Mean annual temperature**
Addis Ababa	1955	29.75	8.04	18.90
Addis Ababa	1960	29.80	7.90	18.85
Addis Ababa	1965	30.36	7.44	18.90
Addis Ababa	1970	30.50	8.80	19.65
Addis Ababa	1975	30.97	8.67	19.82
Addis Ababa	1980	30.79	9.05	19.92
Addis Ababa	1985	22.63	8.82	15.72
Addis Ababa	1990	23.19	9.02	16.10
Addis Ababa	1995	23.73	9.48	16.60
Addis Ababa	2000	23.58	9.66	16.62
Addis Ababa	2005	23.80	9.98	16.89
Addis Ababa	2010	22.87	11.04	16.95
Addis Ababa	2015	24.30	12.80	18.55

**Table 3:** Variation of annual maximum and minimum temperature in *Arba Minch* (1975–2015).

	**Year**	**Average annual temp. (Max)**	**Average annual temp. (Min)**	**Mean annual temperature**
Arba Minch	1985	31.06	18.18	24.62
Arba Minch	1990	30.20	17.95	24.08
Arba Minch	1995	30.36	17.23	23.80
Arba Minch	2000	30.65	16.67	23.66
Arba Minch	2005	30.35	17.40	23.88
Arba Minch	2010	29.99	17.89	23.94
Arba Minch	2015	31.38	17.91	24.65

**Table 4:** Variation of annual maximum and minimum temperature in *Axum* (2006–2016).

	**Year**	**Average annual temp. (Max)**	**Average annual temp. (Min)**	**Mean annual temperature**
Axum	2006	24.30	12.03	18.16
Axum	2007	26.26	12.28	19.27
Axum	2008	26.30	12.18	19.24
Axum	2009	27.37	12.57	19.97
Axum	2010	26.65	12.19	19.42
Axum	2011	26.05	11.70	18.87
Axum	2012	26.04	11.73	18.88
Axum	2013	26.04	11.90	18.97
Axum	2014	25.94	11.58	18.76
Axum	2015	26.59	11.78	19.18
Axum	2016	26.92	12.33	19.62

**Table 5:** Variation of annual maximum and minimum temperature in *Bahir Dar* (2002–2016).

	**Year**	**Average annual temp. (Max)**	**Average annual temp. (Min)**	**Mean annual temperature**
Bahir Dar	2002	26.20	7.3	16.75
Bahir Dar	2003	29.55	12.7	21.13
Bahir Dar	2004	25.56	12.68	19.12
Bahir Dar	2005	26.96	12.92	19.94
Bahir Dar	2006	26.75	12.87	19.81
Bahir Dar	2007	26.78	10.32	18.55
Bahir Dar	2008	26.83	11.59	19.21
Bahir Dar	2009	27.60	12.33	19.96
Bahir Dar	2010	27.07	12.52	19.80
Bahir Dar	2011	26.99	11.46	19.22
Bahir Dar	2012	27.68	12.11	19.85
Bahir Dar	2013	28.78	11.61	20.20
Bahir Dar	2014	27.69	14.08	20.88
Bahir Dar	2015	28.50	13.78	21.17
Bahir Dar	2016	27.02	14.68	20.85

**Table 6:** Variation of annual maximum and minimum temperature in *Beshoftu* (1951–2013).

	**Year**	**Average annual temp. (Max)**	**Average annual temp. (Min)**	**Mean annual temperature**
Beshoftu	1951	25.56	9.95	17.75
Beshoftu	1955	26.27	10.84	18.55
Beshoftu	1960	26.19	11.56	18.87
Beshoftu	1965	26.50	11.69	19.09
Beshoftu	1970	26.34	11.80	19.07
Beshoftu	1975	25.49	11.03	18.26
Beshoftu	1980	26.58	11.28	18.93
Beshoftu	1985	25.96	10.80	18.38
Beshoftu	1990	26.30	11.95	19.13
Beshoftu	1995	26.86	12.43	19.65
Beshoftu	2000	26.72	12.10	19.41
Beshoftu	2005	26.60	10.37	18.48
Beshoftu	2010	26.54	11.02	18.78
Beshoftu	2013	29.45	10.91	20.18

**Table 7:** Variation of annual maximum and minimum temperature in *Combolcha* (1952–2015).

	**Year**	**Average annual temp. (Max)**	**Average annual temp. (Min)**	**Mean annual temperature**
Combolcha	1952	20.98	8.34	14.66
Combolcha	1955	23.15	12.24	17.69
Combolcha	1960	26.80	12.65	19.7
Combolcha	1965	25.90	11.87	18.89
Combolcha	1970	26.00	12.40	19.20
Combolcha	1975	24.99	10.65	17.82
Combolcha	1980	26.38	11.75	19.07
Combolcha	1985	26.05	5.59	15.82
Combolcha	1990	26.50	12.96	19.73
Combolcha	1995	26.38	12.60	19.49
Combolcha	2000	26.80	11.79	19.30
Combolcha	2005	27.25	12.20	19.73
Combolcha	2010	27.49	13.325	20.40
Combolcha	2015	28.11	12.85	20.48

**Table 8:** Variation of annual maximum and minimum temperature in *Debre Markos* (1955–2015).

	**Year**	**Average annual temp. (Max)**	**Average annual temp. (Min)**	**Mean annual temperature**
Debre Markos	1955	21.58	8.90	15.24
Debre Markos	1960	22.25	8.15	15.20
Debre Markos	1965	21.98	8.58	15.28
Debre Markos	1970	22.96	9.94	16.45
Debre Markos	1975	22.40	9.12	15.76
Debre Markos	1980	22.45	10.00	16.22
Debre Markos	1985	21.90	10.21	16.06
Debre Markos	1990	22.57	9.51	16.04
Debre Markos	1995	23.34	10.56	16.95
Debre Markos	2000	22.60	10.09	16.35
Debre Markos	2005	23.05	10.60	16.83
Debre Markos	2010	23.05	11.12	17.09
Debre Markos	2015	23.56	11.18	17.37

**Table 9:** Variation of annual maximum and minimum temperature in *Dire Dawa* (1952–2015).

	**Year**	**Average annual temp. (Max)**	**Average annual temp. (Min)**	**Mean annual temperature**
Dire Dawa	1952	32.58	12.56	22.57
Dire Dawa	1955	31.69	18.03	24.86
Dire Dawa	1960	31.26	18.68	24.97
Dire Dawa	1965	31.22	18.19	24.70
Dire Dawa	1970	31.07	18.79	24.93
Dire Dawa	1975	31.26	18.79	25.02
Dire Dawa	1980	31.20	19.60	25.40
Dire Dawa	1985	31.21	19.14	25.17
Dire Dawa	1990	31.45	19.30	25.37
Dire Dawa	1995	32.36	19.33	25.85
Dire Dawa	2000	32.58	18.79	25.68
Dire Dawa	2005	32.93	19.19	26.06
Dire Dawa	2010	32.65	19.15	25.90
Dire Dawa	2015	33.00	18.43	25.71

**Table 10:** Variation of annual maximum and minimum temperature in *Gode* (1966–2015).

	**Year**	**Average annual temp. (Max)**	**Average annual temp. (Min)**	**Mean annual temperature**
Gode	1966	34.82	21.14	27.98
Gode	1970	35.00	22.63	28.82
Gode	1975	34.80	22.93	28.86
Gode	1980	34.39	23.46	28.92
Gode	1985	34.14	21.95	28.05
Gode	1990	34.62	23.38	29.00
Gode	2000	34.61	23.20	28.91
Gode	2005	35.21	24.24	29.72
Gode	2010	35.56	24.51	30.0
Gode	2015	35.31	24.04	29.67

**Table 11:** Variation of annual maximum and minimum temperature in *Gondar* (1952–2015).

	**Year**	**Average annual temp. (Max)**	**Average annual temp. (Min)**	**Mean annual temperature**
Gondar	1952	25.65	11.90	18.77
Gondar	1955	25.87	11.42	18.65
Gondar	1960	25.46	11.28	18.37
Gondar	1965	26.63	12.55	19.59
Gondar	1970	27.04	12.76	19.90
Gondar	1975	26.71	13.35	20.03
Gondar	1980	26.55	13.40	19.97
Gondar	1984	26.77	13.55	20.16
Gondar	1990	26.7	12.32	19.54
Gondar	1995	27.33	14.37	20.85
Gondar	2000	27.30	11.58	19.44
Gondar	2005	27.46	13.88	20.67
Gondar	2010	28.00	13.45	20.73
Gondar	2015	27.40	13.94	20.67

**Table 12:** Variation of annual maximum and minimum temperature in *Gore* (1952–2015).

	**Year**	**Average annual temp. (Max)**	**Average annual temp. (Min)**	**Mean annual temperature**
Gore	1952	22.16	12.56	17.36
Gore	1955	22.79	12.90	17.85
Gore	1960	23.40	12.94	18.17
Gore	1965	22.35	13.11	17.73
Gore	1970	23.41	13.06	18.24
Gore	1975	22.67	13.14	17.90
Gore	1980	23.44	13.77	18.60
Gore	1985	22.50	13.70	18.10
Gore	1990	25.20	14.70	19.95
Gore	1995	24.56	13.78	19.17
Gore	2000	24.01	13.64	18.82
Gore	2005	24.50	14.62	19.56
Gore	2010	24.20	14.65	19.42
Gore	2015	24.60	14.12	19.36

**Table 13:** Variation of annual maximum and minimum temperature in *Hawassa* (1975–2015).

	**Year**	**Average annual temp. (Max)**	**Average annual temp. (Min)**	**Mean annual temperature**
Hawassa	1975	25.79	11.57	18.68
Hawassa	1980	27.18	12.61	19.90
Hawassa	1985	26.42	11.05	18.74
Hawassa	1990	27.21	12.30	19.76
Hawassa	1995	27.86	12.55	20.20
Hawassa	2000	27.35	12.41	19.88
Hawassa	2005	27.61	12.81	20.21
Hawassa	2010	27.05	14.20	20.62
Hawassa	2015	28.54	14.35	21.44

**Table 14:** Variation of annual maximum and minimum temperature in *Jimma* (1952–2015).

	**Year**	**Average annual temp. (Max)**	**Average annual temp. (Min)**	**Mean annual temperature**
Jimma	1952	25.76	11.25	18.50
Jimma	1955	26.95	10.12	18.54
Jimma	1960	28.28	10.33	19.30
Jimma	1965	26.69	10.95	18.82
Jimma	1970	26.18	11.28	18.73
Jimma	1975	26.39	10.81	18.60
Jimma	1980	27.18	11.20	19.19
Jimma	1985	26.97	10.69	18.83
Jimma	1990	26.52	11.50	19.01
Jimma	1995	28.05	11.83	19.94
Jimma	2000	28.62	10.95	19.79
Jimma	2005	27.90	11.31	19.60
Jimma	2010	27.37	12.91	20.14
Jimma	2015	28.54	12.32	20.43

**Table 15:** Variation of annual maximum and minimum temperature in *Mekele* (1960–2015).

	**Year**	**Average annual temp. (Max)**	**Average annual temp. (Min)**	**Mean annual temperature**
Mekele	1960	24.38	10.87	17.62
Mekele	1965	25.53	11.60	18.57
Mekele	1970	25.67	11.60	18.63
Mekele	1975	22.56	11.41	16.99
Mekele	1980	24.21	11.11	17.66
Mekele	1985	24.12	10.30	17.21
Mekele	1990	22.40	11.20	11.20
Mekele	1995	23.93	11.83	17.88
Mekele	2000	24.46	11.85	18.15
Mekele	2005	24.50	11.52	18.01
Mekele	2010	24.45	12.00	18.23
Mekele	2015	24.58	11.88	18.23

**Table 16:** Variation of annual maximum and minimum temperature in *Metehara* (1985–2015).

	**Year**	**Average annual temp. (Max)**	**Average annual temp. (Min)**	**Mean annual temperature**
Metehara	1985	33.27	16.50	24.89
Metehara	1990	32.97	17.76	25.37
Metehara	1995	33.80	18.55	26.17
Metehara	2000	33.62	17.21	25.42
Metehara	2005	34.06	17.80	25.93
Metehara	2010	33.82	18.32	26.07
Metehara	2015	35.19	18.58	26.88

**Table 17:** Variation of annual maximum and minimum temperature in *Neghele* (1952–2015).

	**Year**	**Average annual temp. (Max)**	**Average annual temp. (Min)**	**Mean annual temperature**
Neghele	1952	25.10	12.22	18.66
Neghele	1955	24.88	11.87	18.37
Neghele	1960	25.67	13.65	19.66
Neghele	1965	25.97	12.25	19.11
Neghele	1970	25.31	13.45	19.38
Neghele	1975	25.35	12.99	19.17
Neghele	1980	27.08	13.01	20.05
Neghele	1985	25.03	13.22	19.12
Neghele	1990	27.46	15.01	21.23
Neghele	1995	26.35	15.89	21.12
Neghele	2000	26.64	14.86	20.75
Neghele	2005	26.33	16.24	21.28
Neghele	2010	27.06	16.46	21.76
Neghele	2015	28.22	16.66	22.44

**Table 18:** Variation of annual maximum and minimum temperature in *Nekemte* (1970–2015).

	**Year**	**Average annual temp. (Max)**	**Average annual temp. (Min)**	**Mean annual temperature**
Nekemte	1970	24.00	10.50	17.25
Nekemte	1975	23.15	10.97	17.06
Nekemte	1980	23.48	12.65	18.06
Nekemte	1985	23.18	12.11	17.65
Nekemte	1990	23.99	13.03	18.51
Nekemte	1995	24.35	13.16	18.75
Nekemte	2000	24.40	12.80	18.60
Nekemte	2005	24.87	13.28	19.00
Nekemte	2010	24.56	13.41	18.99
Nekemte	2015	24.15	12.99	18.57

**Table 19:** Variation of annual maximum and minimum temperature in *Robe* (1962–2015).

	**Year**	**Average annual temp. (Max)**	**Average annual temp. (Min)**	**Mean annual temperature**
Robe	1962	19.65	5.80	12.72
Robe	1965	18.40	5.97	12.19
Robe	1970	19.40	6.47	12.93
Robe	1975	19.69	5.95	12.82
Robe	1980	21.45	6.55	14.00
Robe	1985	21.22	9.01	15.12
Robe	1990	21.61	8.42	15.02
Robe	1995	21.91	7.95	14.93
Robe	2000	21.99	6.18	14.08
Robe	2005	22.45	7.95	15.20
Robe	2010	21.85	9.01	15.43
Robe	2015	22.83	8.60	15.72

## 2 Methods and materials

The unprocessed long-term elements of climate such as rainfall and temperature data obtain from the National Metrological Agency (NMA) of Ethiopia were analyzed using tables and graphic trends of analysis. Annual rainfall and mean annual temperature of 18 representative weather stations were computed in order to calculate the country's mean annual rainfall and the inter-annual fluctuations and average annual temperature. The article used different year agricultural sample survey reports of Central Statistical Agency (CSA) of Ethiopia. Based on the data, the trends of major cereals crop production, area cultivated under improved seeds, local seeds, types of fertilizer and applied areas and the consumer price index in Ethiopia were calculated and presented. The author used Microsoft EXCEL software to analyze the data and present the result in graphs and tables.

