# Supplementary material for: Long-term climate data description in Ethiopia
Source: Data Brief. 2017 Jul 29;14:371–92. doi: 10.1016/j.dib.2017.07.052 (PMC5552378; doi:10.1016/j.dib.2017.07.052)
Supplement: Supplementary file 1 — Transparency document [file mmc1.docx]

**Disclosure statements**

There is no potential conflicting of interest reported by the author.
